# Rapid Avian Diversity Recovery Following Photovoltaic Module Removal: Rebounds in Larger Waterbirds Composition and Habitat Restoration in Lake Littoral Areas

**DOI:** 10.3390/ani16132063

**Published:** 2026-07-04

**Authors:** Lei Cheng, Bingguo Dai, Zhenhua Wei, Shuyue Cheng

**Affiliations:** 1 School of Biological Engineering, Huainan Normal University, Huainan 232038, China; chenglei@hnnu.edu.cn (L.C.); sycheng2022@126.com (S.C.); 2 Anhui Province Key Laboratory of Wetland Ecosystem Protection and Restoration, Anhui University, Hefei 230601, China; 3 Key Laboratory of Bioresource and Environmental Biotechnology of Anhui Higher Education Institutes, Huainan Normal University, Huainan 232038, China; 4 School of Biology and Food Engineering, Hefei Normal University, Hefei 230601, China; 5 College of Chemical and Environmental Engineering, Hanjiang Normal University, Shiyan 442000, China; weizhenhua@hjnu.edu.cn

**Keywords:** waterbird diversity, body size, ecosystem recovery, functional nestedness, photovoltaic module removal

## Abstract

In the context of accelerated progress in clean energy development, an increasing number of regions are utilizing photovoltaic modules (PVMs) for the purpose of energy production. Numerous studies have demonstrated that the installation of these PVMs can exert both positive and negative effects on local ecosystems in different regions. However, there is a paucity of studies that have explored the ecological recovery mechanisms following PVM removal from another perspective, which may hinder a comprehensive understanding of PVM ecological impacts and impede the formulation and implementation of ecological management policies. The PVMs installed on Jiaogang Lake, which were to be removed due to policy adjustments, provided a valuable opportunity to address this issue. The taxonomic and functional diversity of waterbird communities in Jiaogang Lake was assessed and compared before and after the removal of PVMs. The results demonstrated that following the removal, there was a significant increase in both waterbird species richness and functional diversity, whilst the functional nestedness patterns between waterbird communities became more pronounced, implying the restoration of lake littoral areas. Subsequent analysis of individual waterbird functional traits revealed a significant trend towards larger body sizes in waterbirds following the removal of PVMs. Although temporal changes in certain traits did not attain statistical significance, the consistent trends observed in numerous traits led to this extrapolation with confidence. The present study thus aims to encourage decisive decision-making, particularly in short-term macroecological research in the local-scale region, avoiding delays in ecological management measures due to an obsession with perfect statistical data and analysis outcomes.

## Simple Summary

Solar energy facilities using photovoltaic modules (PVMs) are increasingly installed worldwide, yet the ecological consequences of their removal remain poorly understood. In this study, we used the planned removal of PVMs from Jiaogang Lake as a natural experiment to evaluate changes in waterbird communities. We compared waterbird diversity and functional traits before and after PVM removal. Our results showed that waterbird species richness and functional diversity increased significantly after removal, and waterbird communities exhibited stronger functional nestedness, indicating recovery of lake littoral habitats. We also observed a clear shift toward larger-bodied waterbird species following PVM removal, suggesting improved habitat suitability. Even though not all trait changes were statistically significant, the overall consistent trends provide meaningful ecological insights. Our findings highlight that timely ecological management decisions can and should be informed by coherent biological patterns, even when statistical outcomes are not perfectly conclusive.

## 1. Introduction

Amid escalating global environmental and energy crises, authorities worldwide are proactively aligning their strategies with the sustainable development goals proposed by the UN [1]. This effort aims to accelerate the deployment and advancement of clean energy sources [2]. Among these sources, photovoltaic technology has emerged as a pivotal contributor to expanding the share of clean energy due to its environmentally friendly and non-polluting characteristics. The adoption of photovoltaic modules (PVMs) has proven effective in reducing the reliance on fossil fuels, significantly curbing greenhouse gas emissions and mitigating the adverse impacts of climate change [3]. However, due to the spatial requirements of PVM installations, selecting optimal locations demands a careful balance between land use efficiency and land values [4]. To minimize the occupation of high-value land resources while optimizing the use of otherwise unused spaces, PVMs are often deployed in underutilized areas, such as suburban wastelands, hillsides, and other low-value lands, or in littoral waters in lakes, ponds, and reservoirs, etc. [5,6,7,8].

However, it has been hypothesized that such siting may inadvertently trigger changes in ecosystem response. For instance, in arid and semi-arid regions, the implementation of PVMs has been shown to enhance local microclimatic conditions by reducing surface solar radiation and decreasing wind speeds. This, in turn, has led to a recovery in vegetation and an increase in biodiversity [9,10]. The construction of PVMs in the Gobi region has been demonstrated to have a significant impact on biodiversity, with increased plant species richness and biomass being observed [11,12]. In addition, the operation of floating PVMs in aquatic environments has been demonstrated to exert a certain degree of influence on the localized ecology, including alterations to key water quality parameters such as water temperature, dissolved oxygen, total organic carbon, chlorophyll a, etc. [13,14]. These alterations can potentially engender far-reaching consequences for zooplankton and fish habitats [15]. The findings of this study indicate that the PVM is not merely an act of energy exploitation; rather, it may also have far-reaching ecological effects on the ecosystem.

In light of the accelerated development of photovoltaic deployment, most studies focus on the positive or negative impacts of PVM installations on the local ecology and environment [16]. However, there is a lack of answers to questions such as what will happen when they are removed. This paucity of research may result in inaccurate estimations of the ecological impact of PVM installations, which hinders the development of rigorous and efficient ecological conservation policies and measures. Fortunately, the PVM removal project in Jiaogang Lake, eastern China, provides a valuable opportunity to explore the ecological effects of PVMs from another direction. This project, which involves the removal of PVMs originally deployed in the lake’s littoral waters in response to policy adjustments, enables the evaluation of the effects of PVM removal as opposed to their installation, in contrast to earlier research that concentrated on ecological changes following the installation of PVMs [9,10,11,12].

Avian species occupy a high trophic level within wetland food webs, and the diversity and spatial–temporal dynamics of waterbird assemblages closely reflect the structural and functional status of these ecosystems; they can therefore serve as effective bioindicators of wetland ecosystem condition. In recent decades, biodiversity assessment methods have evolved considerably. Conventionally, these assessments were primarily based on taxonomic information concerning species as the fundamental identification unit, with the number of species in a region and their dynamics serving as the primary metric [17]. This approach is both intuitive and simple and has supported ecological researchers in discovering and proposing several important fundamental ecological theories [18,19]. However, it has also been posited that the conventional approach simply treats species with divergent ecological traits in an identical manner, thereby overlooking the diversified responses of disparate organisms to changing environmental conditions [20]. Recently, the functional diversity assessment approach based on species functional traits is regarded as a means of obtaining more comprehensive biodiversity information. In the process of enumerating the species within a given community, the methodology employed entails the incorporation of ecological capabilities and quantifiable functional traits [21,22,23]. These traits encompass, but are not limited to, feeding ability, mobility, and body size. It is noteworthy that these traits are characterized by their high degree of adaptation to the local environment, a process that is developed by individual species over the course of prolonged evolutionary periods [24]. Consequently, this methodology exhibits an enhanced capacity to detect subtle alterations in ecosystemic structures.

The PVMs originally installed in the shallow waters along the lake shoreline occupied part of the waterbird habitat, which is also a major living area for plankton and juvenile fish. Consequently, the removal of PVMs may result in the creation of reestablished habitat space and enhanced food resource availability for waterbirds. This, in turn, may have a significant impact on their taxonomic and functional diversity, as well as their distribution patterns. In this study, we hypothesized that the removal of PVMs in littoral waters would alleviate habitat restrictions, thereby triggering a concurrent increase in both the taxonomic and functional diversity of local waterbird communities. Furthermore, we anticipated that larger waterbird species, previously constrained by the physical presence of PVM structures, would reoccupy these restored habitats more frequently, signaling a broader optimization and recovery of the ecosystem structure. The findings of this study will contribute to the enhancement of the research system for ecological impacts of PVMs, thereby providing a theoretical foundation for the planning and sitting of future photovoltaic projects and ecological management strategies.

## 2. Materials and Methods

### 2.1. Study Area

To rigorously assess the ecological impacts of PVM removal, a Before-After-Control-Impact (BACI) design was implemented. Two adjacent freshwater lakes near the Huaihe River, Jiaogang Lake (Impact site) and Huajia Lake (Control site), were selected as the study areas (Figure S1). Both lakes share a subtropical humid monsoon climate, with a mean annual temperature of 15.0 °C and an average annual precipitation of 905.2 mm [25]. Importantly, they possess highly similar landscape characteristics, aquatic vegetation communities, and elevations, and both serve as critical stopover and wintering habitats for migratory waterbirds along the East Asian–Australasian Flyway.

**Figure 1:**
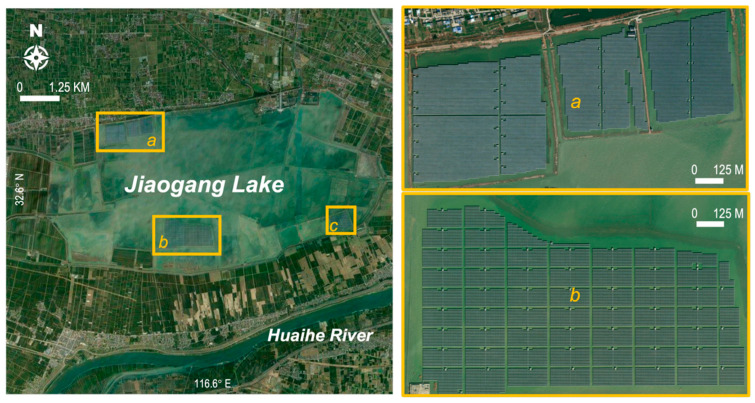
Satellite images of the studied Jiaogang Lake. The three yellow squares (a–c) show the locations where PVMs were installed. Zoomed images of two of these locations are shown on the right.

Jiaogang Lake (32°34′38″–32°37′17″ N, 116°32′06″–116°40′42″ E), located in the north-central part of Anhui Province in East China, with the main stream of the Huaihe River to its north, provides abundant food resources for many waterbird species and serves as one of the most important stopover sites for migratory waterbirds [26]. Jiaogang Lake spans a total water extent that varies from 46.7 km^2^ during the wet season to 37.5 km^2^ during the dry season [25]. Prior to 2023, piling PVM facilities covering a total area of 2.15 km^2^ had been installed along its northern, southern, and southeast shores (Figure 1). Although contributing to clean energy production, these installations were reported to be associated with reduced oxygen availability in local water bodies, declines in plankton diversity and abundance, and elevated nutritional levels for other wetland species. In the summer of 2023, the local government removed all PVM facilities from the wetland area.

In contrast, the adjacent Huajia Lake (spanning approximately 22.8 km^2^) remained completely free from any PVM installations or subsequent removal events during the entire study period. Its ecological and hydrological conditions remained undisturbed, making it an ideal reference site to account for natural interannual fluctuations in waterbird abundances. By establishing Huajia Lake as the control site, this BACI framework ensures that any structural and functional shifts observed in the waterbird communities at Jiaogang Lake between the pre-removal (October 2022 to April 2023) and post-removal (October 2023 to April 2024) periods can be reliably attributed to the habitat restoration driven by PVM removal, rather than regional environmental noise or natural temporal variance.

### 2.2. Waterbird Surveys

We divided the waterbird wintering season into three periods based on seasonal water level fluctuations and migration phenology: early (October–December), middle (January–February), and late (March–April). To evaluate changes in waterbird communities before and after the removal of PVM systems, we conducted surveys during the early, middle, and late periods of each wintering season at five sampling sites across Jiaogang Lake (from October 2022 to April 2023, and October 2023 to April 2024). Meanwhile, parallel surveys were conducted using the identical methodology at four sites in Huajia Lake, where the PVM status remained unchanged throughout the two-year study period.

At each sampling site, a single skilled observer employed the look-see counting method during a standard 30 min observation period to estimate flock sizes on clear days without strong winds or heavy fog [27]. Waterbird species, abundances, and ecological groups within an approximate 1 km radius of each survey point were recorded using binoculars (SWAROVSKI 10 × 42 WB, Absam, Austria) and a monocular telescope (SWAROVSKI 20–60 × 85, Absam, Austria). Specifically, the direct individual counting method was applied when a flock was within 200 m and consisted of fewer than 200 individuals. For larger flocks (>200 individuals) or those at greater distances (>200 m), flock sizes were estimated using the agglomerate counting method [28].

### 2.3. Trait-Based Multidimensional Hypervolume

A total of 12 functional traits were encompassed in assessing functional avian diversity based on the AVONET dataset [29]. These traits include morphological characteristics such as the size of the beak, tarsus, and wing, as well as considerations for ecological preferences (Table 1). Subsequently, the multi-trait Gower distance between each pair of species was calculated, as this approach has been demonstrated to produce a relatively balanced contribution between continuous and categorical traits [30,31]. Thereafter, a three-dimensional kernel density hypervolume was constructed on the basis of the initial three trait vectors, which had been derived from a principal coordinate analysis [32]. The construction of kernel density hypervolume was achieved by employing the BAT package (Version 2.11.1) [33] in R (Version 4.6.0) [34].

### 2.4. Data Analysis

#### 2.4.1. Calculation of TD and FD

Taxonomic alpha diversity was quantified as the waterbird species richness within each sampling site. To measure functional alpha diversity, a multi-trait Gower distance matrix was first constructed from the 12 functional traits, followed by a principal coordinate analysis to extract the primary trait vectors. The volume of a three-dimensional kernel density hypervolume shaped by these initial three vectors was then calculated to represent the functional alpha space of each community [35].

Taxonomic beta diversity was evaluated through pairwise community dissimilarity. Utilizing the abundance-based framework, the total taxonomic dissimilarity was partitioned into two distinct components representing independent ecological processes: turnover, which reflects the balanced substitution of individuals among different species between sites, and nestedness, which denotes the systematic loss of individuals from richer to poorer sites [36]. Functional beta diversity indices, including functional turnover and nestedness, were derived using a similar partitioning approach based on the trait hypervolume overlap. Additionally, community-weighted mean (CWM) values were computed to reflect the dominant functional characteristics of the waterbird assemblages. For continuous morphological characteristics, CWM was calculated as the abundance-weighted average of trait values across co-occurring species, whereas for categorical life-history preferences, CWM was represented by the relative abundance of the focal avian groups.

#### 2.4.2. Statistical Analysis

To rigorously test the ecological effects of PVM removal while controlling for regional environmental variance, diversity metrics were analyzed within a Before-After-Control-Impact (BACI) experimental design. The primary focus of these models was the significance of the two-way Time × Treatment interaction term, which provides statistical confirmation that any observed biological shifts are driven by the localized habitat restoration rather than natural regional interannual fluctuations.

Permutational multivariate analysis of variance (PERMANOVA) was applied to the abundance-based dissimilarity matrices to assess comprehensive community-wide composition shifts across the experimental groups. The PERMANOVA models evaluated the interaction of Time × Treatment using 999 permutations to confirm whether the temporal trajectory of community structures at the impact lake diverged significantly from the control lake. Furthermore, linear regression models were employed to assess the correlation between taxonomic and functional beta diversity during each study phase. A bootstrap resampling procedure with 1000 iterations was performed to evaluate shifts in the slopes of these linear regressions between the pre- and post-removal periods, with a 95% confidence interval excluding zero indicating a significant alteration in community functional redundancy.

We also quantified community-weighted mean (CWM) values for each continuous functional trait following the equation ${CWM}_{jk}=\sum_{i=1}^{n}P_{ik}\times t_{ij}$, where *P_ik_* represents relative abundance of species *i* recorded in community *k*, *t_ij_* means value of functional trait *j* of species *i*, while *n* is the total species richness. The significance of temporal changes in CWM for each continuous functional trait between the pre- and post-removal periods was measured using the Brunner-Munzel test. As for categorical traits, CWM is assessed as the relative abundance of focal avian species.

All diversity metrics and statistical procedures were executed in R [34]. Taxonomic beta diversity partitioning was performed via the betapart package (version 1.4) [36], functional hypervolumes were modeled using the BAT package [33], CWM calculations were conducted with the FD package (Version 1.0-12) [37], the PERMANOVA tests were performed using vegan package (Version 2.7-3) [38], and the bootstrap simulations were implemented via the boot package (Version 1.3-32) [39].

## 3. Results

### 3.1. Taxonomic Diversity Changes

The implementation of the BACI experimental design provided rigorous verification of the ecological effects of PVM removal on waterbird taxonomic alpha diversity. The two-way analysis of variance (ANOVA) revealed a statistically significant interaction effect between Time and Treatment for waterbird species richness (*F*_1,14_ = 5.553, *p* = 0.034; Table 2). While the mean species richness at the undisturbed control site (Huajia Lake) exhibited stable natural baseline patterns across the two wintering seasons, the impact site (Jiaogang Lake) demonstrated a substantial increase from 6.0 ± 2.2 species per site before removal to 10.8 ± 0.7 species per site following PVM removal. The significance of this interactive term explicitly establishes that the observed recovery of taxonomic richness was driven by the localized habitat restoration resulting from PVM elimination rather than regional interannual fluctuations or environmental noise.

### 3.2. Community Composition Shifts and Functional Space Reassembly

To dissect the comprehensive variations in community structures, a permutational multivariate analysis of variance (PERMANOVA) was conducted on the abundance-based Bray–Curtis dissimilarity matrices (Table 3). The results demonstrated a highly significant baseline difference in waterbird composition between the impact and control lakes (Treatment effect: *F*_1,14_ = 8.071, *p* = 0.001), reflecting inherent habitat variations. Crucially, within the BACI framework, the interaction between time and treatment displayed a marginally significant trend (F_1,14_ = 1.829, *p* = 0.100). Given the inherent high temporal variance and spatial heterogeneity typical of macroecological field data, this marginal significance strongly indicates a divergent trajectory, suggesting that while the overall abundance hierarchy of dominant species requires a longer temporal window to completely reorganize, the removal of PVM structures has successfully initiated a targeted shift in community composition at Jiaogang Lake relative to the control site.

**Figure 2:**
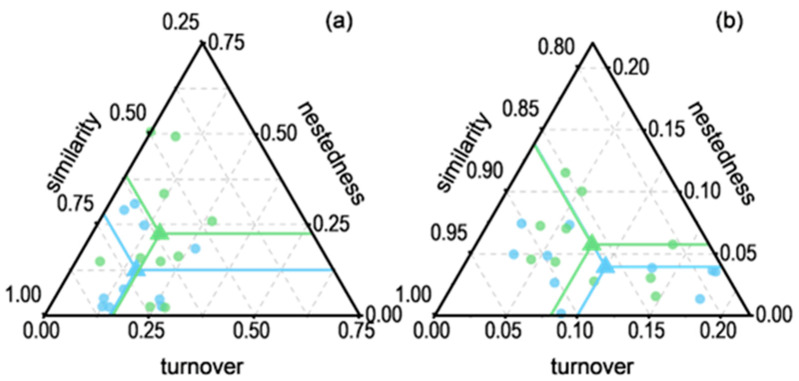
Ternary scatter plots with three taxonomic (**a**) and functional (**b**) indices as axes: similarity, turnover, and nestedness between pairs of waterbird communities, respectively. Blue represents pre PVM removal, and green represents post removal. Each dot in the figure represents a pair of communities, and the triangles represent the average values. The blue and green lines represent the respective three means that determine the positions of the two triangles.

**Figure 3:**
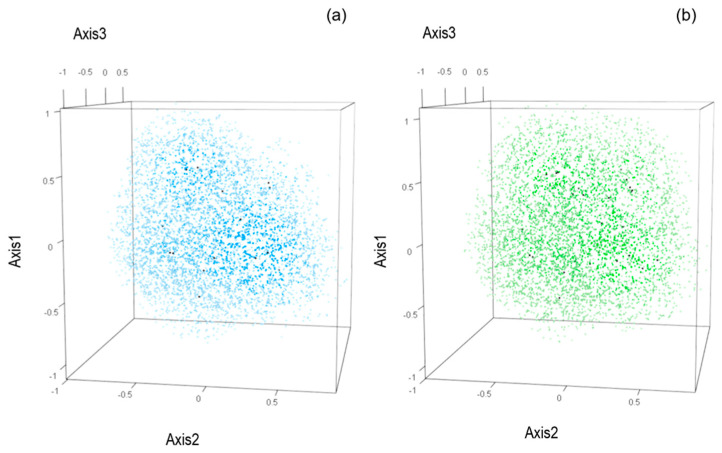
Three−dimensional hypervolumes representing functional space composed by observed waterbird communities pre (**a**) and post (**b**) PVM removal. Black dots denote observed species locations; blue and green point clouds indicate random simulations based on the kernel density model.

To further deconstruct the mechanisms underlying this ongoing transitional shift in community structures, the taxonomic and functional beta diversity patterns were analyzed alongside morphological trait spaces. Concurrently with the richness expansion, the total taxonomic beta diversity index and its partitioned components (turnover and nestedness) did not exhibit statistically significant temporal alterations across the study phases (Figure 2a). However, the multi-dimensional functional space analysis revealed profound reassembly. The three-dimensional kernel density hypervolume derived from core avian functional traits was significantly enlarged following PVM removal, indicating a distinct increase in waterbird functional alpha diversity (Figure 3). While functional turnover remained stable without significant temporal changes (*p* > 0.49), a statistically significant reinforcement of functional nestedness patterns was captured (*p* = 0.03; Figure 2b). This indicates that species with similar ecological capabilities occurred across a wider array of sampling sites after habitat restoration.

### 3.3. Association Between Taxonomic and Functional Waterbird Diversity

**Figure 4:**
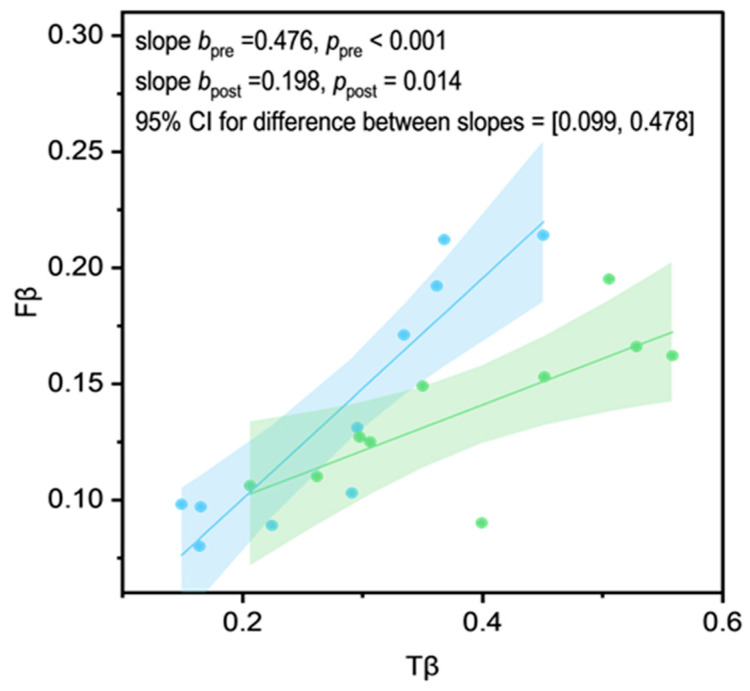
Linear regressions showing the pairwise dissimilarity of waterbird community taxonomic and function pre (blue) and post (green) PVM removal. The slope and significance of the regression models are shown in the figure, respectively. Additionally, the 95% confidence intervals for the differences in slope between the two regressions, obtained through bootstrap simulations, are also presented.

Furthermore, linear regression analysis confirmed positive correlations between taxonomic and functional dissimilarity during both periods (Figure 4). Notably, bootstrap simulations demonstrated a significant decrease in the regression slope following PVM removal (95% CI = [0.099, 0.478]; Figure 4). This declined slope implies that identical degrees of species dissimilarity yielded lower functional divergence post-removal, meaning that similar functional traits were distributed across more sites, which mathematically supports the strengthening of the functional nestedness pattern.

### 3.4. Shifts in Essential Functional Traits

**Figure 5:**
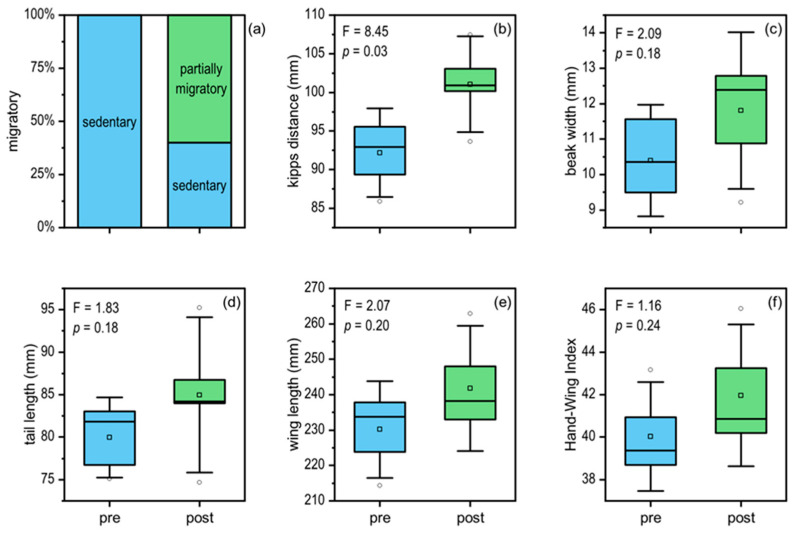
Boxplots showing CWM of (**a**) migratory, (**b**) kipp’s distance, (**c**) break width, (**d**) tail length, (**e**) wing length, and (**f**) Hand-Wing Index of waterbird communities pre (blue) and post (green) the PVM removal.

These multi-faceted diversity shifts were tightly coupled with directional changes in community-weighted mean (CWM) values of key functional traits. Regarding life-history preferences, sedentary waterbirds exclusively dominated the functional composition across all communities prior to PVM removal; post-removal, partially migratory waterbirds with more complex life histories expanded their presence, dominating more than half of the recorded assemblages (Figure 5a). Simultaneously, the CWM index values for continuous morphological traits reflecting waterbird body size—including the Hand-Wing Index, beak width, wing length, and tail length—exhibited a consistent upward trend following PVM removal (Figure 5c–f). Among these, a statistically significant increment was confirmed for kipp’s distance (*F* = 8.45, *p* = 0.03; Figure 5b). Taken together, these coherent biological shifts reveal a clear transition toward larger-bodied waterbirds and more intricate migratory life histories, validating the progressive optimization of the restored littoral ecosystem.

## 4. Discussion

In summary, the findings of the present study demonstrate that following the removal of PVM structures along the littoral waters of Jiaogang Lake, there was a significant increase in waterbird diversity. This increase was reflected not only in greater species richness but also in a significant expansion of their functional space. The positive correlation between taxonomic and functional richness is consistent with the findings of many biodiversity studies [40,41], suggesting that an increase in species richness indicates that local habitat conditions are conducive to the preservation of a greater number of species. This is indicative of enhanced habitat recovery and diversity, thereby satisfying the functional requirements of species described by diverse ecological characteristics [42]. Furthermore, following the removal of PVMs, a significant increase in the functional nestedness patterns of waterbird communities was observed, indicating that species with similar functions occurred at more sites, thereby providing the ecosystem with higher functional redundancy [40]. Functional redundancy is defined as the phenomenon in which multiple species within an ecosystem perform analogous ecological functions, thereby reducing the impact of the extinction of a single species on ecosystem functions. For instance, in the context of climate change or human-induced disturbances, certain species may undergo population declines due to habitat loss or resource scarcity [43]. However, the presence of functional redundancy enables other species to fill their niches, thereby maintaining the overall stability of the ecosystem [44]. Consequently, functional redundancy, a pivotal indicator of ecosystem stability, merits heightened consideration in prospective ecological restoration and conservation initiatives.

In Jiaogang Lake, the PVM was originally installed in the shallow water area in proximity to the shore. These areas, characterized by elevated water temperatures, sluggish currents, and an inhospitable environment for large predatory fish, constitute the predominant habitat for plankton and juvenile fish [45]. It has been determined that this location offers an optimal environment for a significant population of avian species, notably those of a larger stature and those that typically engage in wading behavior. The installation of PVMs had a significant impact on the avian community, affecting both their access to food sources and the utilization of space, particularly by larger waterbirds. Conversely, following the removal of the PVMs, the abundance of food resources resulted in the restoration of coastal habitats for various waterbird species, including large waterbirds, thereby promoting a rebound in waterbird diversity.

The trends in CWM values for various functional traits after PVM removal also corroborate this ecological process. CWM values for multiple functional traits characterizing waterbird body size, including kipp’s distance, increased after PVM removal, indicating that larger waterbird species are gradually occupying a more significant position in the functional structure of waterbird communities. Furthermore, a comparison of pre- and post-removal periods revealed a shift in waterbird community composition, with the presence of predominantly resident waterbirds at all observation sites before the removal of the PVMs, contrasting with the subsequent dominance of partially migratory waterbirds with more complex life histories. This observation also suggests that the presence of the PVMs may impede the migratory movements of avian species, thereby potentially compromising the significant ecological function of Jiaogang Lake as a migratory waterbird stopover site.

Although both taxonomic and functional diversity assessment methods revealed significant temporal changes in waterbird community alpha diversity, only the functional diversity assessment framework based on functional traits was able to detect temporal changes in nestedness patterns. Similar phenomena have been observed in biodiversity studies of other taxa [46]. A notable example of this is the fish communities that have been observed in lakes situated within the middle and lower reaches of the Yangtze River. While the taxonomic diversity of fish communities did not demonstrate a significant response to alterations in water level, the incorporation of data pertaining to the five core ecological functions of fish indicated that the functional structure of fish communities had undergone significant changes [47,48]. The examination of the functional characteristics of species in direct interaction with their local environment is a key component of functional diversity pathways. This approach enables ecological researchers and conservation practitioners to gain precise insights into the functional mechanisms underlying biodiversity changes and the environmental changes they reflect [20]. The results of this study demonstrate an increasing trend in CWM values for multiple functional traits characterizing waterbird body size after PVM removal. This suggests that the existence of PVMs limits the accessibility of large waterbirds to lake littoral waters and indicates that PVM removal has restored waterbird habitats to a certain extent. The validity of such inferences is challenging to substantiate with classical taxonomic diversity results. The functional diversity approach is advantageous in that it combines species ecological functions with environmental adaptability, thereby enabling ecological researchers and conservation practitioners to precisely understand the functional mechanisms underlying biodiversity changes [49]. Moreover, the functional diversity approach has the capacity to furnish significant references for the assessment of ecosystem service functions [50]. Consequently, the functional diversity approach has considerable application prospects in ecological research and conservation practices.

While the findings highlight a rapid recovery of waterbird diversity, the inherent data collection challenges of macroecological field surveys must be acknowledged [51]. Significant environmental heterogeneity across different observation sites combined with the limited one-year post-removal temporal window can introduce substantial variance [52,53,54], which is often exacerbated by unpredictable seasonal or hydrological fluctuations [45]. Such field constraints can easily obscure strict statistical significance at the conventional level for certain individual metrics [55,56]. To account for these field-data characteristics, robust non-parametric methodologies, such as the Brunner-Munzel test, were utilized to flexibly handle data heterogeneity and potential outliers [57]. More importantly, rather than relying solely on isolated *p*-values, this study emphasizes the integration of multiple indicators to synthesize overall biological trends [58]. Although interannual variations in specific traits did not always reach traditional statistical significance levels, the coherent and directional biological shifts, most notably the distinct transition toward larger-bodied and partially migratory waterbirds, provide robust evidence for habitat restoration. This comprehensive approach demonstrates that meaningful ecological inferences can be reliably drawn from short-term assessments by prioritizing consistent biological patterns alongside adaptable statistical frameworks.

The findings of this study carry significant implications for the decision-making process concerning local ecological management measures. Firstly, it is imperative to make prompt adjustments to conservation policies based on the findings of scientific research to prevent the loss of opportunities for ecological restoration [59]. However, local institutions often face constraints imposed by political factors, including official terms of office and career development, which hinder the formulation of ecological management policies [60,61]. This, in turn, impedes the ability to make scientific decisions based on long-term observation results. Consequently, researchers are advised to enhance collaboration with local institutions by offering scientific evidence and policy recommendations to facilitate the timely implementation of ecological restoration measures. Secondly, this study emphasizes the value of small-scale, short-term research in the context of ecological management decision-making [62]. Despite the challenges posed by limited data and statistical inference difficulties, the findings of such studies can serve as valuable references for local ecological restoration initiatives. For instance, in the present study, while the temporal changes in certain functional traits did not attain statistical significance, the overall trend indicated that the elimination of PVM had a favorable effect on the functional structure of waterbird communities. This information is of great significance for the timely and decisive formulation of targeted ecological conservation measures. Consequently, future ecological research should place greater emphasis on collaboration with local institutions, leveraging the practical application of scientific research to promote the effective implementation of small-scale ecological management measures.

## 5. Conclusions

This study provides compelling empirical evidence that the removal of photovoltaic modules (PVMs) from the littoral waters of Jiaogang Lake facilitated a rapid and significant recovery in local waterbird communities. By employing a rigorous Before-After-Control-Impact (BACI) experimental design, we demonstrated that the habitat restoration post-removal directly drove increases in both taxonomic species richness and functional diversity. Notably, the waterbird assemblages exhibited enhanced functional nestedness and a distinct compositional shift toward larger-bodied and partially migratory species, signifying the progressive restoration of littoral ecosystem structures and the return of vital stopover functions. Methodologically, this study highlights the efficacy of incorporating trait-based functional diversity metrics alongside traditional taxonomic approaches, particularly for detecting rapid ecological shifts in short-term field surveys. Ultimately, these findings underscore the critical importance of timely and decisive ecological management. They demonstrate that conservation policies, when informed by consistent multidimensional biological patterns, can successfully reverse human-induced habitat constraints and restore ecosystem functionality.

## Figures and Tables

**Table 1:** Functional traits encompassed in functional diversity assessment of waterbird communities.

Trait Code	Variable Type	Morphological Characteristic/Category
BLC	Continuous	Beak length from tip to skull along culmen
BLN	Continuous	Beak length from tip to nares
BW	Continuous	Beak depth
BD	Continuous	Beak width
TL	Continuous	Tarsus length
WL	Continuous	Wing length
KD	Continuous	Kipp’s distance
SD	Continuous	Secondary distance
HWI	Continuous	Hand-Wing Index
MG	Categorical	Migration type: Sedentary; Short-distance migratory; Long-distance migratory
TL	Categorical	Trophic level: Carnivorous; Herbivorous; Omnivorous
PL	Categorical	Primary lifestyle: Aquatic; Terrestrial; Generalist

**Table 2:** Two-way analysis of variance (ANOVA) testing the effects of time (pre- vs. post-removal), treatment (impact vs. control site), and their interaction on waterbird species richness.

Source of Variation	*df*	Sum of Squares	*F*-Value	*p*-Value
Time	1	32.00	4.23	0.059
Treatment	1	354.02	46.78	<0.001 ***
Time × Treatment	1	42.03	5.55	0.034 *
Residuals	14	105.95		

Note: Significance levels are indicated as * *p* < 0.05, *** *p* < 0.001.

**Table 3:** Permutational multivariate analysis of variance (PERMANOVA) testing the effects of time, treatment, and their interaction on waterbird community composition based on Bray–Curtis dissimilarity.

Source of Variation	*df*	Sum of Squares	*R* ^2^	*F*-Value	*p*-Value
Time	1	0.10	0.03	0.85	0.518
Treatment	1	0.98	0.33	8.07	0.001 ***
Time × Treatment	1	0.22	0.07	1.83	0.100
Residuals	14	1.70	0.57		
Total	17	3.01	1.00		

Note: Significance levels are indicated as *** *p* < 0.001.

## Data Availability

The raw data supporting the conclusions of this article will be made available by the authors on request.

## Supplementary Materials

The following supporting information can be downloaded at: https://www.mdpi.com/article/10.3390/ani16132063/s1, Figure S1: The relative position of Jiaogang Lake and Huajia Lake.
