# Supplementary figures and images for: Rapid Avian Diversity Recovery Following Photovoltaic Module Removal: Rebounds in Larger Waterbirds Composition and Habitat Restoration in Lake Littoral Areas
Source: Animals (Basel). 2026 Jul 4;16(13):2063. doi: 10.3390/ani16132063 (PMC13359581; doi:10.3390/ani16132063)

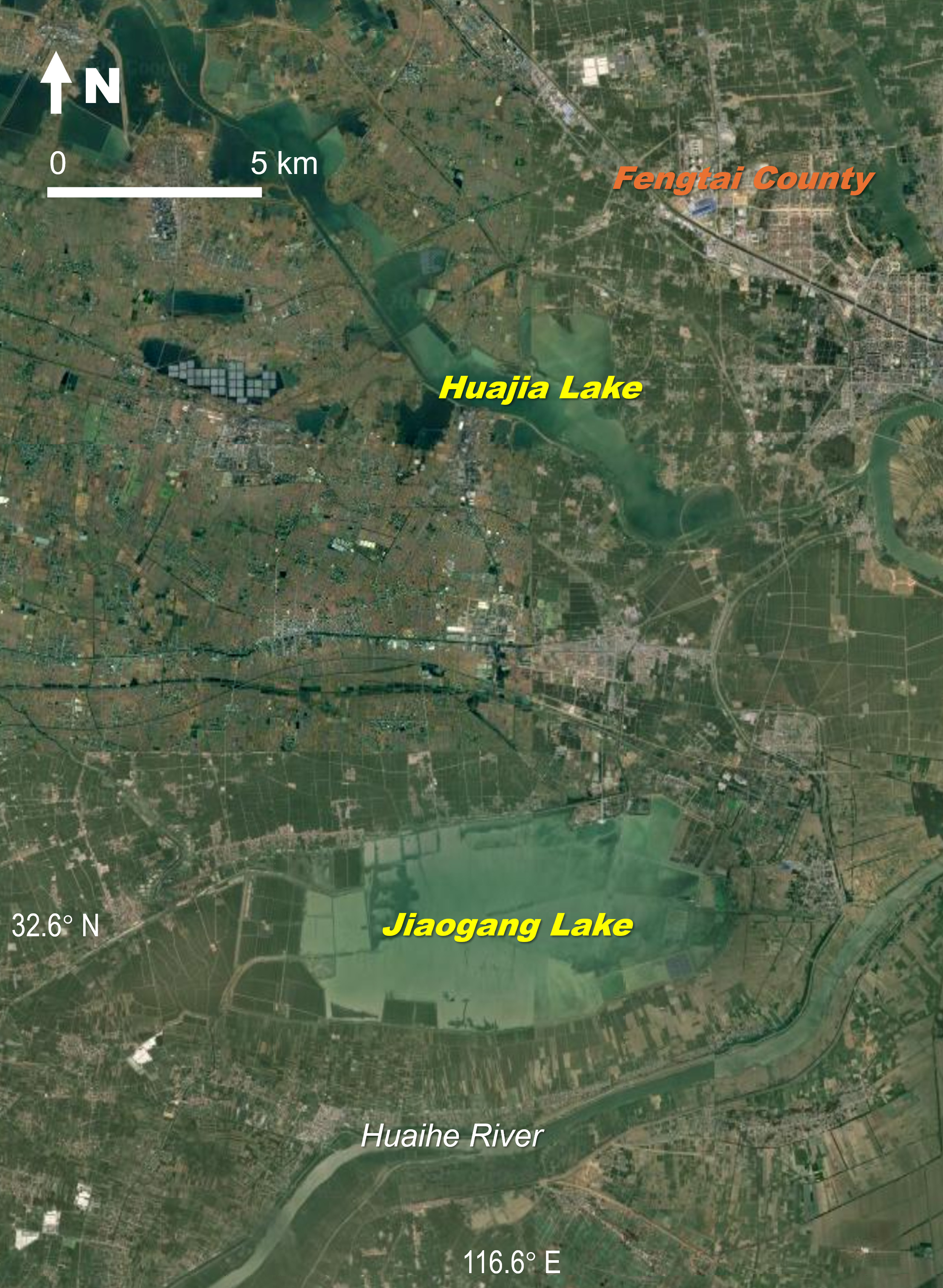

Supplement: Supplementary file 1 [file animals-16-02063-s001.zip › animals-4385634-supplementary.jpg]
